# Assessment of Isokinetic Trunk Muscle Strength and Fatigue Rate in Individuals after Bariatric Surgery

**DOI:** 10.3390/medicina60040534

**Published:** 2024-03-26

**Authors:** Nouf H. Alkhamees, Olfat Ibrahim Ali, Osama R. Abdelraouf, Zizi M. Ibrahim, Aya Abdelhamied Mohamed

**Affiliations:** 1 Department of Rehabilitation Sciences, College of Health and Rehabilitation Sciences, Princess Nourah bint Abdulrahman University (PNU), Riyadh 11671, Saudi Arabia; nhalkhamees@pnu.edu.sa; 2 Physical Therapy Program, Batterjee Medical College, Jeddah 21442, Saudi Arabia; olfat.sayed@bmc.edu.sa (O.I.A.); pt4.jed@bmc.edu.sa (O.R.A.); 3 Department of Biomechanics, Faculty of Physical Therapy, Cairo University, Giza 12613, Egypt; aya.abdelhamied@pt.cu.edu.eg

**Keywords:** obesity, trunk strength, bariatric surgery, fatigue, EMG, isokinetic dynamometer

## Abstract

*Background and Objectives*: Lean body mass loss after bariatric surgery (BS) is remarkable, despite an effective long-term mass reduction and significant declines in comorbidities. A person’s functional capacity is adversely affected when their skeletal muscle strength declines by up to 30%. This study aimed to assess the isokinetic trunk muscle strength and fatigue rate in individuals after BS. *Materials and Methods*: This study included fifty-eight patients, both male and female, ranging in age from 19 to 45. Twenty-seven individuals had BS and twenty-seven healthy people served as the control group. The primary outcomes were the measurement of the concentric and eccentric isokinetic muscle strength of the trunk flexor and extensor muscles. An isokinetic dynamometer (Biodex Rehabilitation and Testing System 3) was used for the assessment of the isokinetic muscle strength. Noraxon EMG was used to determine a secondary outcome, which was the median frequency slop (MF/time) and root mean square slop (RMS/time) of the lumbar erector spinea muscle at 50% of the Maximum Voluntary Isometric Contraction (MVIC). Outcome measures were assessed for both groups. *Results*: Compared to the control group, the bariatric group showed a lower mean value of both concentric and eccentric isokinetic muscle strength for the flexor and extensor trunk muscles (*p* < 0.05). In terms of the EMG fatigue rate, the RMS slope increased significantly more than that of the control group, while the MF slope decreased (*p* > 0.05). *Conclusions*: The current study found that, in comparison to the healthy subjects, the BS group showed reduced levels of fatigue and isokinetic strength in the trunk muscles. Based on these results, it is recommended that individuals who underwent BS take part in tailored rehabilitation programs to avoid potential musculoskeletal issues in the future.

## 1. Introduction

Obesity is a chronic condition resulting in the excess accumulation of body fat, often in the abdominal area, which results in multiple increased health risks. Obesity is associated with various musculoskeletal disorders, including impairment of the spine [1]. A weight loss of 5–10% of body weight is frequently advised to combat obesity-related complications and enhance the quality of life of those who are obese [2]. An integrated strategy is needed to effectively manage obesity, encompassing not only medical procedures but also dietary modifications and improving physical inactivity [3]. When standard weight loss therapy through lifestyle changes fails, bariatric surgery (BS) is a treatment option to be taken into consideration [4]. A gastric bypass (Roux-en-Y), laparoscopic sleeve gastrectomy (LSG), and adjustable gastric band (AGB) are the three primary types of bariatric procedures that are performed. These procedures all function a little bit differently. More recent statistics show that, while AGB has been declining recently, SG is currently becoming more popular [5]. The results of a systematic review that comprised 36 studies and 2570 patients indicated that LSG is a safe and effective procedure for initial weight loss [6]. Furthermore, a retrospective cohort study found that, compared to a gastric bypass, LSG was linked to a lower long-term risk of mortality and postoperative complications [7].

More substantial weight loss happens due to dietary restriction and surgically induced nutritional malabsorption [8]. According to Sczepaniak et al., 6 months after an LSG or GB, the comparable percentages of total weight loss (%TWL) were 26.8% and 24.0%, and after 12 months, they were 29.5% and 34.2% [9]. In addition to reducing fat mass, bariatric surgery also reduces muscle and bone mass, or “fat-free mass” (FFM). This decline in FFM has negative effects on patients’ health because it increases the risk of fractures, reduces muscle strength, and impairs their functional abilities [10,11]. A recent systematic review and meta-analysis concluded that, within a year after surgery, there was a loss of over 8 kg of LBM and skeletal muscle mass, with most of this loss occurring within the first three months after surgery. The study highlighted the importance of having interventions in place to lessen these losses [12].

Several assessment instruments have been used in the past to examine the strength of the muscles in the upper and lower limbs following bariatric surgery [13,14,15,16]. The isokinetic strength of leg muscles in the post-bariatric population was first measured in a study by Santos et al. [17]. Unfortunately, no previous studies have assessed the isokinetic strength of the trunk muscles following BS. Trunk muscle strength is important in manual handling and other activities of daily living such as lifting and rising from a seated position [18]. These muscles are involved in key mobility tasks and are activated at approximately 30% of their maximal voluntary strength values during walking [19]. Low back pain (LBP) has been linked to trunk muscle strength. Muller et al. [20] found an association between chronic LBP and decreased lumbar extensor isokinetic strength. A retrospective study reported that subjects after BS experienced a new episode of low back pain (LBP) [21].

Low energy and easy fatigability are commonly reported among patients with chronic illnesses. Low back pain is also predicted to occur due to poor back extensor muscle endurance [22]. As a result, a large body of research has concentrated on using power spectral analysis of the electromyographic (EMG) signal to quantify back muscle endurance impairments [23].

The most widely used indicators of muscle fatigue are the rate of the decrease in median frequency (MF) and the simultaneous rise in EMG amplitude (RMS) during submaximal sustained contraction [24]. Using McGill’s torso muscular endurance tests, Ali et al. [25] found that trunk muscle endurance decreased after BS in comparison to their controls.

The findings of previous studies showed inconsistent results about the assessment of muscle strength after BS. Although many studies revealed that severe weight loss brought on by bariatric surgery (BS) resulted in a decline in muscle strength [14,26,27], Reinmann et al. found that subjects who underwent BS did not experience changes in either power or muscle strength [28]. Furthermore, a recent systematic review concluded that BS is a useful weight-loss strategy that does not sacrifice muscular strength [29]. Moreover, no previous research has used EMG to investigate the back extensor fatigue rate. Therefore, our study aimed to assess the isokinetic trunk muscle strength and fatigue rate after bariatric surgery. It was hypothesized that individuals after BS will experience a decline in both isokinetic trunk strength and the EMG fatigue rate.

## 2. Materials and Methods

### 2.1. Study Design

This cross-sectional study was carried out between March 2023 and 20 October 2023 at the college biomechanical lab. The study was approved by the Ethics Committee of Princess Nourah bin Abdulrahman University (IRB Log Number: 23-0232) in compliance with the ethical guidelines outlined in the Declaration of Helsinki.

### 2.2. Subjects

The present study included 58 participants in total, ranging in age from 19 to 45. The two groups were the bariatric and control groups. The bariatric surgery group consisted of 30 individuals (8 males and 22 females) who were at least six months post-bariatric surgery. According to reports, the period of most rapid and significant weight loss following bariatric surgery was the first six months, during which time skeletal muscle mass was also lost [30]. In order to recruit participants, health professionals at several bariatric surgery centers identified patients who had undergone weight reduction surgery for obesity at least six months prior to the study recruitment phase using databases and clinic lists. These patients received general information about the study, and those who were interested got in touch with the lead investigator.

The interested subjects were screened for eligibility criteria to be included in the study. The following were considered as exclusion criteria: involvement in a structured physical activity program, history of surgery, visceral or systemic diseases, spinal disorders, nerve root compression, lower limb injury, in addition to pregnancy in women. Twenty-eight healthy individuals served as a control group (eight males and nineteen females) and were matched in terms of demographic data with the BS group; the control group enrollment was conducted through advertisement. To match the bariatric surgery group, subjects who met the criteria for being overweight (BMI ranges from 25.0 to <30) and sedentary were placed in the control group.

After being informed about the purpose and methods of the study, each participant filled out an informed consent form. Sample size calculation was performed using G*Power 3.1 (Universities, Dusseldorf, Germany) software. Level of significance was 5%, power was 80%, and minimal effect size was 0.8. Isokinetic strength of the trunk muscle was considered the primary outcome for a minimum of 54 patients in both groups.

### 2.3. Outcome Measures

The primary outcome, trunk muscle isokinetic strength, was measured for both the study and control groups. Subsequently, the secondary outcome, the back muscles’ EMG fatigue rate, was assessed. Both assessments were completed on the same day, separated by a 30 min break to avoid the impact of fatigue. Before the results of each assessment were actually recorded, one familiarization trial was allowed.

### 2.4. Measurements

#### 2.4.1. Isokinetic Trunk Muscle Strength Assessment

The Biodex System 3 Pro (Biodex Medical Systems, Inc., Shirley, NY, USA) was used to conduct the isokinetic concentric and eccentric trunk strength test. It has been noted that this apparatus is a dependable means of evaluating strength [31]. The subject was placed in a flexed position with their hands and arms crossed over their thorax. Three sets of five maximal sequential concentric lumbar flexions and extensions at a speed of 60°/s were included in the measurement. A verbal encouragement was given to each person to give it their all during the assessment. Measurements of isokinetic muscle strength were conducted using the Al-Shenqiti et al. [32] protocol. This protocol was recommended by Estrázulas et al. [33] as the most reliable method for evaluating trunk flexors and extensors. This protocol involves testing trunk muscle strength while seated at velocities of 60°/s.

#### 2.4.2. Fatigue Rate Assessment

Noraxon Desktop DTS (Noraxon, Scottdale, PA, USA; sampling frequency: 1500 Hz; filter: low pass 500 Hz) was used to measure the myoelectric activity [34]. Bilateral surface electromyography electrodes were placed over the longissimus muscle (medial lumbar erector spinae) at the L3 level. Compared to the lateral muscles, the medial muscles were found to be more fatigable (VanDieen JH et al.) [35]. More importantly, compared to the lateral muscles, the medial muscles produce higher reliability results (Larivière C et al.) [24]. The reference electrode was placed over the right anterior superior iliac spine (ASIS). The Fast Fourier Transform was applied to the data within the window to determine a power spectrum density.

Using the Biodex system, the subject carried out three maximal isometric efforts from standing, lasting six seconds each, in order to determine the maximal voluntary contraction (MVC). The percentage load during fatigue testing was determined using the best of the three MVCs with two minutes of rest in between trials. Yang et al. [36] concluded that the standing position demonstrates acceptable convergent validity (r = 0.50) in correlation analyses, whereas the sitting position demonstrates relatively low validity (r = 0.32). The fatigue test was one 60 s test conducted at 50% MVC [37].

The magnitude of the electromyographic signal was quantified by the RMS value, whereas the electromyographic spectral content was evaluated by the MF value of the power spectra (fast Fourier transform, Hanning window processing). RMS and MF were calculated from a succession of 21 windows (250 ms) equally spaced from the 5th second to the 55th second of the 60 s contraction and was averaged for both sides. We applied a least squares linear regression analysis to the RMS and MF time series to calculate the rate of decline in MF over time (MF/time slope) and the rate of increase in RMS over time (RMS/time slope), which are indicative of muscle fatigue. Both EMG indices were shown to be valid and reliable for assessment of muscle fatigue [23,24].

### 2.5. Statistical Analysis

All statistical measures were performed using IBM SPSS Statistics Version 25 for Windows. Data were screened for normality assumption through using Kolmogorov–Smirnov normality tests, and histograms and box plots were drawn to check for the presence of extremes. Concentric and eccentric peak torque isokinetic muscle strength for both flexor and extensors trunk muscles, MF slop, RMS slop, and numerical demographic data show non parametric distribution so the Mann–Whitney U test was used to compare theses outcomes between the BS and control groups. The Chi square test was used to compare the nominal data between groups. The level of significance was set at *p* < 0.05 for all comparisons.

## 3. Results

The characteristics of the groups are presented in Table 1. There are no between-group significant differences concerning age, weight, height, BMI, and sex as *p* values were (>0.05), as shown in Table 1.

### 3.1. Concerning Isokinetic Trunk Muscle Strength

In the BS group the mean values for the concentric flexor, eccentric flexor, concentric extensors, eccentric extensors were 149.88 ± 20.8, 135.3 ± 20.58, 137.2 ± 25.64, and 126.4 ± 25, respectively, while in the control group, these values were 167.77 ± 25.67, 156.4 ± 25.18, 182 ± 21.83, and 167.19 ± 21. The results revealed that the concentric and eccentric isokinetic peak torque of both the flexor and extensors muscle groups were lower in the BS group compared to the control group (*p* = 0.001 & 0.001, <0.001, <0.001), respectively, as is shown in Table 2. These results indicate that the BS group has a lower trunk strength in comparison with control group.

### 3.2. Concerning EMG

The MF slop and RMS values during 60 s of 50% MIVC were −0.43 ± 0.22 and 0.27 ± 0.11 in the BS group, while these values were −0.29 ± 0.16 and 0.19 ± 0.08 in the control group. A significant back fatigue was observed among individuals after BS, as the MF slop negatively declined and RMS slop was significantly increased in the BS more than the control group (*p* = 0.006 and 0.002), respectively. These findings indicate that the BS group suffered from a higher fatigue rate of the trunk muscle.

## 4. Discussion

The current study’s results confirmed the research hypothesis that, in comparison to normal controls, individuals who underwent bariatric surgery (BS) showed significantly lower isokinetic back muscle strength and lower endurance, as indicated by the higher EMG fatigue rate. It is well known that decreased trunk muscle endurance and strength are among the risk factors that are strongly linked to a higher likelihood of developing sciatica, or lower back pain [38] Routine assessment of these risk factors could help avoid cases of low back pain after BS. Moreover, a systematic review conducted by De Blaiser et al. concluded that lower extremity injuries may be associated with deficiencies in different aspects of trunk stability [39].

The isokinetic dynamometer is the gold standard instrument for muscle strength assessment (1). The adopted protocol for isokinetic testing of trunk muscle strength in a sitting position at a velocity of 60°/s was reported by Estrázulas et al. [40] to have excellent reliability (ICC = 0.93). The protocol included assessments of both concentric and eccentric trunk strength because eccentric muscle contractions play a crucial role in maintaining trunk stability and are involved in different aspects of core function and movement [41].

The significant postoperative loss of lean body mass (LBM) in individuals after BS may be responsible for the decrease in the isokinetic peak torque of the trunk flexors and extensors. According to Nuijten et al. [12] this loss is predominant within the first 3 months post-surgery and continues for up to 6 months. A previous meta-analysis by Haghighat et al. revealed that, depending on the procedure, LBM losses following bariatric surgery could vary from 17.5% to 31.3% of the overall weight loss [42]. Weight loss and LBM loss are, therefore, strongly correlated, with greater weight loss inevitably leading to greater LBM loss. Because muscle is essential for many body functions, including bone strength, metabolic health, and functional ability, an excessive loss of muscle tissue may have negative long-term effects [12]. According to research by Voican et al. [43], within a year after bariatric surgery, the predictive score for sarcopenia rose from 8% to 32%. This would raise the risk for mortality, frailty, functional impairment, and cardiometabolic disorders when combined with a decrease in muscle strength [44].

Unfortunately, no prior research has examined the trunk muscle strength in the post-bariatric population, so it would be difficult to compare our findings with those of others. However, the present study’s findings are within the same context as those concluded by Carvalho et al. [17] who investigated the effects of a 16-week supervised exercise program on patients’ chances of developing sarcopenia following BS. One of the outcome measures, peak torque of the knee flexors and extensors, was measured at baseline (prior to surgery), pre-intervention, and post-intervention (five months following BS). The control group in Carvalho’s study did not engage in any kind of exercise program, just like the participants in our study. Five months after surgery, the control group’s peak knee extensor and flexor torques decreased by 30.2% and 25.7%, respectively.

Similar findings were reported by Oppert et al. [45] who found that, six months after BS, the control group of the study experienced a decline in the lower extremity one-repetition maximum (leg press) of 30.4 kg and a decline in the upper extremity one-repetition maximum (chest press) of 6.2 kg in absolute values. Furthermore, prior research revealed statistically significant decreases in the absolute muscle strength of the lower limbs six and 36 months following surgery [26,46].

On the other hand, the findings of the present study are in contrast with those of Coral et al. [30] who, despite the decrease in skeletal muscle mass shown by bioelectrical impedance analysis, reported increased muscle strength six months following BS in comparison to pre-operative measurement. This contradiction comes from the difference in muscle strength assessment methods. While Coral et al. chose absolute hand grip strength (HGS) as a predictor for general body strength status, our study used an isokinetic machine to measure trunk muscle strength. Moreover, the study by Coral et al. [30] has several limitations, such as a high dropout rate, the use of absolute HGS rather than relative HGS, and a lack of control over physical training and nutritional supplementation post operation.

According to the results of a recent systematic review by Jung et al. [29] strength was not compromised by bariatric metabolic surgery, even though there were reductions in body composition parameters, such as muscle mass. The eleven studies that make up this review have the same flaw: they use HGS as a muscle strength indicator. In particular, HGS may be acknowledged as the recommended screening test in sarcopenia diagnostic guidelines [47], but it is not a direct indicator of strength in a particular muscle group. Therefore, it is recommended that more studies be conducted in the future to measure muscle strength in post-bariatric subjects using direct and objective methods.

Fatigue is one of the main components of vitality, which encompasses both physical and mental aspects of health and is usually assessed in most quality-of-life surveys [48]. The EMG findings of the current study showed fatigue impairments of the lumbar paraspinal muscles in the BS group presented in the form of an increased RMS slope and a decrease in the MF slope.

The recruitment of additional motor units with a higher firing rate to handle prolonged submaximal loading may be the cause of the gradual increase in the RMS slope. On the other hand, a nearly linear decrease in MF indicates that the power density spectrum of the EMG signal is compressed towards the lower frequencies as the contraction is sustained [49]. The reorganization of the activity pattern of distinct paraspinal muscle segments, particularly the lower spinal segments that become vulnerable during to prolonged contractions, in reaction to the functional loads is aimed at preserving spinal stability, while also exhibiting concurrent deficits in endurance [50]. The redistribution of muscle activity between the superficial and deep trunk muscles provides an additional explanation for the EMG signal changes. This happens because of the quick loss of LBM after BS, primarily in the form of reduced glycolytic muscle fibers [51].

Moreover, Daniels et al. [52] reported many neurological adaptations including increased rate-coding, motor unit recruitment and type II fiber activation, and the reduced co-activation of antagonist muscles that happen in the early phases of the resistance training program. Given that our study’s BS group did not engage in any type of resistance training following surgery, it is expected that they lacked these adaptations. Our findings contradict the conclusions of two systematic reviews by Driscoll et al. [53] and Kolotkin et al. [54], which found that obese people who underwent BS reported better levels of fatigue and energy. Nevertheless, a significant flaw in earlier research on fatigue in this population that was included in the previously mentioned systematic reviews is the use of retrospective questionnaires. It is important to consider some of the drawbacks of utilizing self-report questionnaires. Potentially invalid responses could be the primary drawback of self-report questionnaires (recall biases). In addition, response bias is a phenomenon in which respondents might give false information when answering the questions [55]. Furthermore, there was variation in the methods used to complete these questionnaires, in person or online, with or without the assessor present, which could have an impact on the validity of the responses.

The limitations of the current study included that the decreased isokinetic strength and the EMG changes that indicate the easy fatigability of the lumbar paraspinal muscles were not correlated with the percentage of total weight loss after BS. The literature pertaining to these associations has reported contradictory results [56,57]. Furthermore, since all types of BS were covered in the study, there was no comparison made between the common types of weight loss surgery in terms of isokinetic trunk strength and lumbar paraspinal fatigue. Finally, surface EMG electrodes were used to pick up the activity of the lumbar paraspinal muscles, with a possible change in electrode placement during the fatigue protocol.

## 5. Conclusions

The current study found that, in comparison to the healthy subjects, the BS group showed reduced levels of Fatigue resistance (endurance) and isokinetic strength in the trunk muscles. Based on these results, it is recommended that individuals who underwent BS take part in tailored rehabilitation programs to avoid potential musculoskeletal issues in the future.

## Figures and Tables

**Table 1:** Demographic and clinical data of the participants.

Variables	BS Group	Control Group	*p* Value
	Mean ± SD Median (Interquartile Range)	Mean ± SD Median (Interquartile Range)	
Age (years)	35.1 ± 8.5	32.9 ± 8.76	0.627
	37 (10)	38 (9)	
Weight (kg)	83.22 ± 7.8	81.44 ± 8.55	0.358
	84.5 (11)	80 (10)	
Height (cm)	168.1 ± 9.43	169.46 ± 7.2	0.748
	167 (17)	170 (13)	
BMI (kg/m^2^)	29.54 ± 2.2	28.4 ± 3.4	0.072
	29.2 (3)	28.3 (2.51)	
Gender			
F (%)	22 (73.3%)	19 (67.9%)	0.647
M (%)	8 (26.7%)	9 (32.1%)	
Hypertension	4 (13.3%)	5 (17.9)	0.454
DM	2 (6.7%)	3 (10.7%)	0.467
Medication of comorbidities	4 (13.3%)	6 (21.4%)	0.320
Duration after BS	8.17 ± 1.66	-	-
Surgery type			
Bypass surgery	7 (23.3%)		
Sleeve	23 (76.7%)		

SD: standard deviation, *p*-value: significance level, kg: kilogram, cm: centimeter, %: percentage, F: female, M: male, DM: Diabetic Mellitus.

**Table 2:** Comparison of the mean values of isokinetic and EMG parameter outcomes between bariatric and control groups.

Variables	BS Group Mean ± SD Median (Interquartile Range)	Control Group Mean ± SD Median (Interquartile Range)	MD (95% CI)	*p* Value
Concentric flexor (Nm)	149.88 ± 20.8	167.77 ± 25.67	17.8 (5.11–30.65)	0.001
	150 (25) *	173 (26) *		
Eccentric flexor (Nm)	135.3 ± 20.58	156.4 ± 25.18	21.1 (8.55–33.67)	0.001
	140 (17) *	162 (28) *		
Concentric Extensor (Nm)	137.2 ± 25.64	182 ± 21.83	44.8 (31.8–57.82)	<0.001
	142 (39) *	183 (21) *		
Eccentric Extensor (Nm)	126.4 ± 25	167.19 ± 21	40.8 (28.2–53.43)	<0.001
	128 (31) *	168 (13) *		
MF/time	−0.43 ± 0.22	−0.29 ± 0.16	0.14 (0.24–0.03)	0.006
	−0.44 (0.19)	−0.24 (0.22)		
RMS/time	0.27 ± 0.11	0.19 ± 0.08	0.08 (0.03–0.14)	0.002
	0.27 (0.14) *	0.21 (0.10) *		

Nm: Newton meters, MD: mean difference, CI confidence interval, SD: standard deviation, *p* value: significant level at <0.05, MF: Median frequency, RMS: root mean squared. * median (interquartile range).

## Data Availability

Data is available upon request.
